# Decoupling of Plant Growth and Accumulation of Biologically Active Compounds in Leaves, Roots, and Root Exudates of *Hypericum perforatum* L. by the Combination of Jasmonate and Far-Red Lighting

**DOI:** 10.3390/biom11091283

**Published:** 2021-08-27

**Authors:** Martina Paponov, Manya Antonyan, Rune Slimestad, Ivan A. Paponov

**Affiliations:** 1 Norwegian Institute of Bioeconomy Resesarch (NIBIO), Division of Food Production and Society, P.O. Box 115 NO, 1431 Ås, Norway; martina.paponov@outlook.com; 2 Department of Agronomy, Armenian National Agrarian University, Yerevan 0009, Armenia; antonanmana1998@gmail.com; 3 PlantChem AS, Eikenveien 334, 4596 Eiken, Norway; Rune@plantchem.com; 4 Department of Food Science, Aarhus University, 8200 Aarhus, Denmark

**Keywords:** *Hypericum perforatum* L., jasmonate, far-red light, growth–defense dilemma, plant growth, secondary compounds, hypericin

## Abstract

The plant hormone jasmonic acid (JA) fine tunes the growth–defense dilemma by inhibiting plant growth and stimulating the accumulation of secondary compounds. We investigated the interactions between JA and phytochrome B signaling on growth and the accumulation of selected secondary metabolites in *Hypericum perforatum* L., a medically important plant, by spraying plants with methyl jasmonate (MeJA) and by adding far-red (FR) lighting. MeJA inhibited plant growth, decreased fructose concentration, and enhanced the accumulation of most secondary metabolites. FR enhanced plant growth and starch accumulation and did not decrease the accumulation of most secondary metabolites. MeJA and FR acted mostly independently with no observable interactions on plant growth or secondary metabolite levels. The accumulation of different compounds (e.g., hypericin, flavonols, flavan-3-ols, and phenolic acid) in shoots, roots, and root exudates showed different responses to the two treatments. These findings indicate that the relationship between growth and secondary compound accumulation is specific and depends on the classes of compounds and/or their organ location. The combined application of MeJA and FR enhanced the accumulation of most secondary compounds without compromising plant growth. Thus, the negative correlations between biomass and the content of secondary compounds predicted by the growth-defense dilemma were overcome.

## 1. Introduction

The most promising technology for the production of high-value and medicinal plants is protected agriculture (e.g., vertical farming, greenhouse), as this allows for the precise control of environmental conditions at a relatively low production cost (compared with in vitro culture). Hydroponic greenhouse cultivation can also facilitate the harvesting of the aboveground plant parts; however, more importantly, it can also provide access to plant roots and root exudates. The main advantage of harvesting root exudates is that the collection is non-destructive, so important metabolites can be collected over the lifetime of plants. Biologically active chemicals that are exuded into a hydroponics solution can be isolated far more easily than they can from the plant tissues themselves (tissue processing typically requires solvent extraction). The controlled growth conditions used in protected agriculture also reduce the problem of the heterogeneity of the plant’s chemical composition induced by environmental factors. Thus, in combination with a selection of specific genotypes, controlled environmental conditions help in the production of plant products with foreseeable quality (e.g., predictable concentrations of biologically active compounds), thereby meeting a major challenge faced by the pharmacology industry in meeting the desired standardization of herbal-derived products [1].

Surprisingly, the cultivation of high-value medicinal plants in protected agriculture has not received significant attention, partly because of the absence of a clear protocol for maintaining appropriate environmental conditions that enhance both plant growth and the accumulation of biologically active compounds. These two processes are often diametrically opposed, as stress conditions that reduce plant growth in turn enhance the accumulation of secondary products to defend against that stress. This response is termed the growth–defense dilemma [2].

Optimal production of secondary plant products therefore requires that the growth–defense dilemma can be resolved. Plant responses to abiotic and biotic stresses are usually explained by competition for fixed carbon resources between growth (e.g., cell division, cell enlargement, photosynthesis) and defense (e.g., accumulation of secondary defense compounds) [3]. Secondary metabolites play a crucial role in plant adaptation to biotic and abiotic stresses, and they also represent the key ingredients that define the beneficial and/or healing effect of herbs, medicinal, and other high-value plants with effects on human health [4,5]. Therefore, resolving the growth–defense tradeoff is important for the development of sustainable agriculture (i.e., combining high yield with high resistance of crops to unfavorable conditions) and the cultivation of high-value horticultural and medicinal plants with high contents of nutritionally and medicinally significant secondary compounds.

A previous study on Arabidopsis showed that the growth–defense dilemma could be overcome by the simultaneous activation of JA signaling and deactivation of phytochrome B (phyB) signaling pathways [6]. This investigation showed that the addition of a phyB mutation to a mutant that already had five deactivated JAZ repressor proteins (jazQ) resulted in a recovery of plant growth to the wild-type level, but the accumulation of anthocyanins was maintained. Thus, the JA and phyB signaling pathways, acting in concert, might be able to resolve the growth–defense dilemma. PhyB is a reversible photoreceptor that is activated by red and inactivated by far-red light [7]; therefore, the regulation of the growth–defense tradeoff might occur through modulation of the JA pathway and by adjustment of the R and FR light spectrum.

JA is one of the main inducers of stress responses in plants. According to the current response model, the key players in JA signaling are JAZ proteins, which are inhibitors of JA signaling. At low cellular JA concentrations, these proteins bind to MYC transcription factors and block their activity [8]. As the JA concentration increases, JA binds to COPI receptors and induces the ubiquitination and subsequent degradation of the JAZ proteins. This degradation in turn derepresses the MYC transcription factors, which then inhibit plant growth and stimulate a defense response [9].

JA plays a key role in plant responses to different biotic and abiotic stresses, and it strongly modulates the accumulation of secondary compounds [10,11]. For this reason, JA is one of the most widely used elicitors in in vitro culture for the enhancement of the accumulation of medicinally or nutritionally useful compounds in different high-value plants [12].

JA activity interacts with light signaling, where phytochrome signaling pathways play a key role [13,14]. Specifically, Phytochrome A (phyA) plays a key role in the FR high-irradiance response of etiolated seedlings [15] by enhancing the JA-induced inhibition of growth responses and by promoting the expression of JA biosynthesis genes [13]. PhyB also regulates JA activity by effects on both biosynthesis and JA signaling. Deactivation of the phyB signaling pathway reduces JA biosynthesis due to the inhibition of a sulfotransferase (ST2a) that decreases the available pool of precursors for the synthesis of the active forms of jasmonates [16]. This deactivation also reduces the abundance of DELLA proteins that normally stabilize JAZ proteins, thereby ultimately inhibiting JA signaling [17]. The inhibition of JA activity by the deactivation of phyB signaling causes a reduction in the accumulation of secondary compounds [18]. This metabolic shift negatively impacts plant resistance to herbivores and pathogens while also reducing the content of health-promoting compounds in plant materials destined for human use.

Deactivation of phyB, which occurs in response to a low R:FR ratio, also induces the shade avoidance response [15], which is characterized by the rapid elongation of stems and petioles, reduced leaf thickness, and reduced branching [19,20,21]. Jasmonate and FR light have antagonistic effects on both growth and the accumulation of secondary compounds, raising a question about their effects on these processes when applied simultaneously. The answer to this question should further contribute to our understanding of the interplay between JA and phytochrome signaling pathways.

The overcoming of the growth-defense dilemma in Arabidopsis assumes that the negative correlation between plant growth and the accumulation of secondary compounds might also be resolved in other plant species. The resolution of this dilemma is especially important for medicinal plants, as their accumulated secondary compounds are the main reason for their cultivation. One of the most important medicinal plants worldwide in terms of its phytochemistry is St. John’s wort (*Hypericum perforatum*; Hypericaceae). The accumulation of specific and common secondary metabolites at high concentrations makes this plant attractive in pharmacology.

The characteristic compounds accumulated in *H. perforatum* are the naphthodianthrones hypericin and pseudohypericin [22]. Another important group is the flavonoids, which are phenolic secondary metabolites synthesized by a combination of the phenylpropanoid and polyketid pathways, and conjugated to sugars, e.g., glucose, rhamnose and rutinose. The flavonoids include flavones, flavonols (quercetin, kaempferol), and glycosides (rutin, hyperside, and isoquercitrin). Other phenolic compounds, such as caffeic acids and chlorogenic acids, also accumulate in *H. perforatum* [22]. Secondary compounds accumulate in different plant organs [23], and some of these compounds are exuded by the root system into the rhizosphere [24]. However, no comprehensive study has yet examined the distribution of biologically active compounds in different organs and in root exudates of *H. perforatum*.

The aim of the present study was to investigate the effects of JA and FR and their interactions on plant growth, plant-growth-related traits, accumulation of valuable metabolites, and metabolite exudation by roots in the pharmacologically important plant *H. perforatum*. Previous investigations have shown that the plant defense response can be decoupled by the constitutive activation of JA and the deactivation of the phytochrome pathway. This discovery motivated us to hypothesize that a combination of the external application of JA and the switching of the phytochrome signaling pathway by exposure to a low R:FR ratio would also decouple growth and the accumulation of high-value secondary compounds.

## 2. Materials and Methods

### 2.1. Plant Material and Experimental Performance

Seeds of St. John’s wort (*Hypericum perforatum*; Hypericaceae), purchased from Rarexoticseeds (https://www.rarexoticseeds.com/, accessed on 26 February 2018) were sterilized in 2.5% sodium hypochlorite for 10 min and then washed thoroughly 5 times with deionized water. The sterilized *H. perforatum* seeds were germinated in “sandwich” filter paper placed between mat layers (Clas Ohlson, Insjön, Sweden) and fixed in plastic plates. Seeds were sown at 5 mm intervals in a line 2–3 mm below the top of the filter paper and incubated in a 10% full nutrient solution containing 500 µM KNO_3_. The full nutrient solution contained 1 mM CaSO_4_, 1 mM K_2_HPO_4_, 1 mM KH_2_PO_4_, 2 mM MgSO_4_ [25], and micronutrients with the following concentrations: 15 µmoL Fe, 10 µmoL Mn, 5 µmoL Zn, 30 µmoL B, 0.75 µmoL Cu, and 0.5 µmoL Mo. For the first 2 days, the seeds were kept in darkness at 18 °C. Seedlings were transferred individually at 36 days after sowing (DAS) from the “sandwich” system to aerated 0.8 L plastic pots containing 50% nutrient solution and 2.5 mM KNO_3_ and aeration and fixed with foam slabs onto the pot lid. The pots were covered with light-impermeable foil.

### 2.2. Growth Conditions

During the whole cultivation period, the conditions in the growth chamber were maintained a 16 h/8 h day/night photoperiod (1:00–9:00 dark), 22 °C/18 °C day/night temperature, 80/90% day/night air humidity, and atmospheric CO_2_ concentrations. From 45 DAS onward, all plants were cultivated in full nutrient solution. The pH of the nutrient solution was monitored regularly and controlled between pH 5.6 and 6.9. The nutrient solution was replaced on a weekly basis and always before the methyl jasmonate (MeJA) applications. The nutrient solution was continuously aerated.

### 2.3. Experimental Setup

The experimental setup included two far-red (FR) treatments: full light spectrum and minimal addition of FR (Control) and full light spectrum and high addition of FR, in combination with two MeJA treatments: with MeJA treatment and without. In total, we investigated a full factorial experiment with the treatments Control, FR, MeJA and MeJA + FR with six replications per treatment.

### 2.4. Lightning Conditions and FR Supply

The intensity of the top lighting was provided by Heliospectra LED RX30 lamps (Göteborg, Sweden) adjusted to 280 µmoL m^−2^ s^−1^ PAR, with 8 of 9 diodes given the same adjustments for all treatments. For the diodes 370, 400, 420, 450, 530, 630, and 5700 K (white light), the adjustments were the same (20% of the maximum Heliospectra LED RX30 intensity) for all treatments. We also reduced the 660 nm diode to 10% of maximum intensity. For the control treatment, the 735 nm (FR) diode was adjusted to 10%, whereas this diode was adjusted to 100% of the maximum intensity for the high FR treatment.

In addition to the increased FR-supply from the top lighting, the high FR treatment also included light from with one double-sided 2 × 35 W FR lamp with a peak wavelength of 720–740 nm (Barel, Kirkenes, Norway, http://www.barel.no/, accessed on 1 May 2021). The FR lamps were placed at the level of the plant height in each high FR treatment at a distance of 25 cm, so that each plant received additional FR light from one side. Under the control treatment, the reversible phyB photoreceptor is converted by red light into a far-red light (FR)-absorbing form, which is active. The addition of supplemental FR light converts phytochrome B into the R-absorbing form, Pr, which is inactive [26].

### 2.5. Application of MeJA

One day after the seedling transfer from the “sandwich” system to 0.8 L plastic pots (37 DAS), all seedlings were sprayed 4 times with a mock solution (3% ethanol in water, *v*/*v*) for the control or with 10 µM MeJA (392707, Merck, Darmstadt, Germany) in 3% ethanol for the MeJA treatment. Airborne diffusion of MeJA to adjacent plants was prevented by covering all plants with pots, which were only removed after the tiny droplets sprayed on the leaves’ surfaces had dried.

This external application of MeJA to the shoots was continued in the same way throughout the growth period. The plants received MeJA applications at 37, 45, 52, 59 DAS and a final one given to half the plants at 64 DAS and to the other half at 65 DAS.

### 2.6. Root Exudate Collection

The root exudates were collected on two subsequent days (64 DAS, 65 DAS) from half the plants on each day. Three plants of each treatment (Control, MeJA, FR, and FR + MeJA) were transported to pots covered by light-impermeable foil and containing 650 mL continuously aerated distilled water. The plants were then given their respective treatments and left overnight (8 h) and for the next 5 (65 DAS) or 6.5 (64 DAS) daylight hours for root exudate collection and purification. The plants were then transferred to full nutrient solution and sampled.

### 2.7. Final Sampling

The phenology of the plants was recorded before the final sampling by measuring the lengths of all secondary branches of the longest primary branch starting from the plant base.

Plant material (about 2 g fresh weight; FW) was immediately frozen in liquid nitrogen in screw-capped Falcon tubes and vacuum lyophilized for 36 (leaves) and 24 (roots) h in a BK-FD10S freeze-dryer (BIOBASE, Jinan, China) device. The leaf material was powdered (Star-Beater VWR with 5 mm metal balls, 29 Hz for 3 min) to fine dust and stored at −80 °C until further processing.

For analysis of the growth response, the FWs of leaves, roots, and stems were determined. A further 0.3–0.5 g of root material was collected, weighed, and preserved in 50% ethanol for further analysis of root hairs. Specific leaf area was determined by weighing all the leaves from one representative side branch. Images of the leaves were captured with a NIKON d750 camera. The leaves were dried to a constant weight in a ventilated oven at 45 °C, and dry weight was used to determine the specific leaf area of the plants. The individual leaf areas and total leaf area were estimated using Fiji software version (https://imagej.net/software/fiji/, accessed on 27 January 2019).

### 2.8. Analysis of Roots and Root Hairs

The root samples were mounted in water and visualized with an Olympus CX-41 microscope (Olympus Corporation, Tokyo, Japan) and dark-field illumination. Images were captured with an ocular mounted Toupcam U3CMOS 5.1 MP camera (ToupTek Europe, Stansfield, United Kingdom) using ToupView 3.7 software. The average density (hairs/mm) and length (mm) of the root hairs were determined by Fiji software. The dataset is based on measurements of 142–163 root hairs per treatment.

### 2.9. Extraction of Bioactive Compounds from Leaves and Roots

A 100 mg sample of lyophilized and powdered (29 Hz for 3 min Starbeater, VWR, Radnor, PA, USA) leaves and roots of *H. perforatum* were vortexed for 20 min at maximal speed in 2 mL Eppendorf tubes containing a 5 mm diameter steel bead and 1.5 mL 80% methanol. The extract was centrifuged for 5 min at 17,000× *g* and the supernatant collected. The supernatant was centrifuged again to prevent later sedimentation. The clean supernatant was stored at −20 °C until it was assayed for total phenolic content and analyzed by UHPLC.

### 2.10. Collection of Root Exudates

The water containing root exudates was prefiltered using a Sigma-Aldrich^®^ vacuum filtration assembly (Z290432-1EA, Merck, Darmstadt, Germany) and Nalgene bottle-top sterile filters (45 mm diameter and 0.45 μm pore size) (Z370533, Merck, Darmstadt, Germany). Approximately 600 mL of the filtered water-exudate solution was loaded onto Bond Elut™ C18 (Agilent Technologies, Santa Clara, CA, USA) solid-phase extraction cartridges with a 1 g bed mass and 40 µm particle size to trap the non-polar and semi-polar secondary compounds. Columns were activated with 2 mL 100% MeOH (10516279, Fisher scientific, Waltham, MA, USA) followed by 2 mL 1% aqueous formic acid (33015, Fluka, Honeywell, Morris Plains, NJ, USA). The columns were washed with 2 mL distilled water, and the hydrophobic compounds were eluted with 2 mL of 2% formic acid in MeOH. The eluent was stored at −20 °C until the total phenolic and UHPLC analyses.

### 2.11. Assay of Total Phenolic Content

Total phenolic content was estimated in leaves, roots, and root exudates of *H. perforatum* using the Folin–Ciocalteu assay [27]. Freeze-dried samples of 100 mg of DM leaf and 20–100 mg of DM root, as well as 200 µL of concentrated root exudates, were extracted by use of 1.5 mL 80% methanol in darkness for 24 h at 35 °C, then centrifuged for 5 min at 13,000× *g* (Micro Star 17R, VWR, Radnor, PA, USA). The supernatant was collected and diluted 50× and 25× for leaves and roots, respectively, whereas the root exudate was assayed undiluted.

For the analysis, 100 µL of root or leaf extracts or 200 µL of the root exudate solution were combined with 200 µL of a 10% Folin–Ciocalteu (F–C) reagent (F9252, Merck, Darmstadt, Germany). An 800 µL volume of a 700 mM Na_2_CO_3_-solution (S7795, Merck, Darmstadt, Germany) was added, and the samples were incubated at room temperature for 2 h in darkness. Triplicate 100 µL samples were then transferred to a spectrophotometric plate reader (Multiscan GO, Thermo Fisher Scientific, Waltham, MA, USA), and the absorbance was measured for each well at 765 nm at room temperature. Measurements were standardized against gallic acid (48630, Merck, Darmstadt, Germany) (50 μM–2.5 mM in 80% MeOH). The root exudation rate of total phenolics into the 650 mL water–exudate mix was calculated based on the total gallic acid equivalents measured in the 200 µL concentrated extract fraction. The rate of exudation was expressed as the amount of total phenolics per FW of roots and the duration time of exudation.

### 2.12. Quantitative Determination of Phenolic Compounds by UHPLC

Phenolic compounds were analyzed by UHPLC (1290 Infinity II, Agilent Technologies, Santa Clara, CA, USA) with a diode array detector and an electrospray ionization single-quadrupole detector (6120 SQ, Agilent Technologies, Santa Clara, CA, USA). Separation was achieved on an Ascentis Express C18-column (100 × 2.1 mm, 2 µm, Supelco, Merck, Darmstadt, Germany). A gradient with increasing content of acetonitrile (solvent B) in 0.02% formic acid (solvent A) was used as follows: from 2% to 5% (in 1 min), from 5% to 33% (in 6 min), from 33% to 95% (in 10 min), from 95% to 100% (in 3 min), and finally from 100% to 2% (in 1 min). Column recondition was obtained by a post-time of 2 min. The flow rate was set to 0.3 mL/min, and injections were 10 µL. All samples were filtered (0.45 µm) prior to analysis. Mass spectra were acquired in scan mode (180–700, *m*/*z*) with a scan time of 500 ms, fragmentor at 50 V, and both positive and negative modes of ionization. The source was operated with a gas temperature at 300 °C, gas flow at 7.0 L/min, nebulizer at 30 psi, and a capillary voltage at ±3 kV.

Compounds were characterized based on co-chromatography with authentic samples and by their UV-Vis absorbance spectra, as well as by their pseudo-molecular and fragment ions, according to previous reports [28,29,30]. Four acyl phloroglucinols (APG), including hyperfirin, were detected in root extracts. No naphtodianthrones (NDAs) were detected in root extracts. A total of eight NDAs, including hypericin, were detected in exudates. Three NDAs, including the main compound hypericin, were detected in leaf extracts. The NDA content is reported to be the sum of all NDAs for each sample.

Quantifications were made based on the UV-Vis absorbance at the detection windows of 280, 320, 360, and 590 nm for catechins, hydroxycinnamic acids/APG, flavonols, and NDAs, respectively. Standard curves were prepared for each group of phenolic compounds using (–)-epicatechin (Merck, Darmstadt, Germany) for catechin, epicatechin and procyanidin dimer; chlorogenic acid (Merck, Darmstadt, Germany) for chlorogenic acid and coumaroylquinic acid; rutin and isoquercitrin (PlantChem, Eiken, Norway) for flavonols; and pseudohypericin and hypericin (Merck, Darmstadt, Germany) for those compounds. Hypericin was also used for the quantification of APGs at 320 nm.

### 2.13. Quantitative Determination of Pigments, Sugars, and Ions

The extraction of these compounds was based on the one-step extraction described by Salem [31]. This approach is further described in Paponov et al. in 2021 [32] and uses 20 +/− 1 mg freeze-dried leave material from *H. perforatum* for the quantitative determination of pigments and analysis of nonstructural carbohydrates. The extraction of starch also followed the described protocol, but the supernatant required further dilution 10 times with deionized water before assaying for glucose.

Leaf ion composition was analyzed by ion chromatography with conductive detection, as described in Paponov et al. [33]. Prior to analysis, the extracted (semi)polar phase from the one-step extraction was diluted 20-fold and 50-fold with deionized water for cations and anions, respectively, and filtered through 0.45 µm PVDF (hydrophilic) syringe filters (CH2225-PV, Thermo Fisher Scientific, Waltham, MA, USA), discarding at least 1 mL of the first filtrate. Data were blanked and statistically analyzed.

### 2.14. Statistics

Data were statistically analyzed by analysis of variance (two-way ANOVA). The treatments were replicated six times. When significant treatment effects were indicated by ANOVA, Fisher’s protected LSD test was used to compare the individual means (Statistica 13 software package, Palo Alto, CA, USA).

## 3. Results

### 3.1. Plant Growth Traits

**Figure 1:**
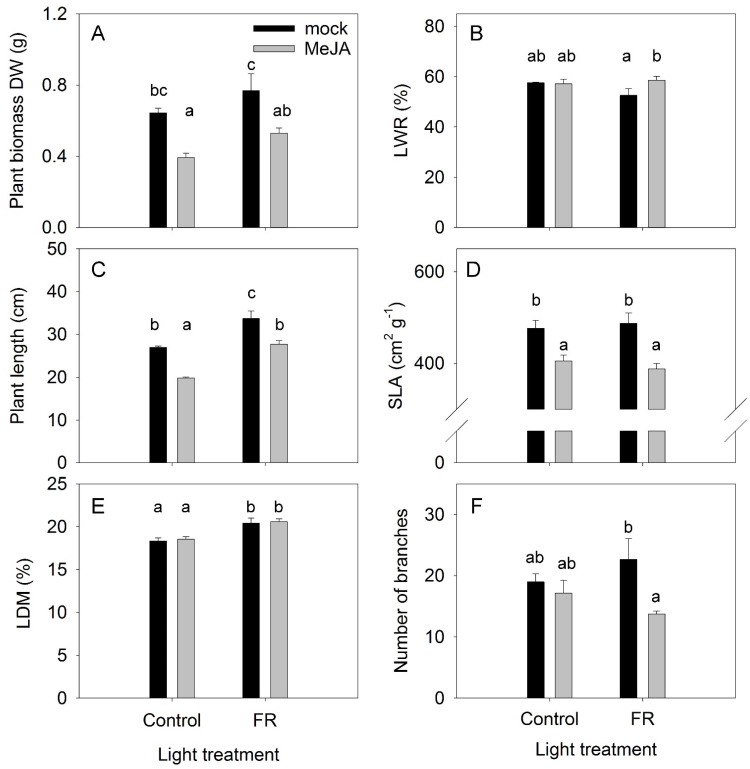
The effect of far-red light and methyl jasmonate (MeJA) application on plant biomass (**A**) leaf weight ratio (LWR) (**B**) plant length (**C**) specific leaf area (SLA) (**D**) leaf dry matter content (LDM) (**E**) and number of secondary branches (**F**) in *Hypericum perforatum* (*n* = 6). Differences between means with different letters are statistically significant. *H. perforatum* seedlings were given different light and hormone treatments at 36 days after sowing (DAS). The final plant samples were taken at 65 DAS.

The effects of the plant hormone MeJA and FR light and their interactions were determined in a full factorial experiment, where every factor was studied at two levels. The effects of MeJA treatment and FR light on plant growth were found to be diametrically opposed: MeJA inhibited and FR increased plant growth (Figure 1A). No interaction was found between MeJA and FR, indicating that the activity of MeJA was independent of FR effects, and vice versa (Table S1). Under the control light condition, the difference in biomass between plants treated with MeJA and the mock treatment was not related to the dry matter partitioning to the leaves, as no differences were observed in leaf weight ratio (LWR) among these treatments. The lowest LWR was observed for FR-treated plants without MeJA application (Figure 1B), indicating that the highest biomass for this treatment was reached despite the lowest dry matter allocation to the leaves among the treatments. In agreement with the effect on plant biomass, MeJA inhibited and FR stimulated plant length without any interaction between these factors (Figure 1C, Table S1). The stimulation of stem elongation by FR is a typical response of plants to supplemental FR lighting [34].

The inhibitory effect of MeJA on plant biomass was related to decreased specific leaf area (SLA) (Figure 1D), a key indicator of leaf construction costs, indicating that a decline in SLA might contribute to the reduction in plant growth. This effect is in agreement with the expected effect of JA on SLA [35], leaf trichome density, and cuticle thickness [36]. However, the enhanced effect of FR on growth was not related to a modulation of SLA, indicating that a higher photosynthesis rate per unit leaf area might be the reason for the more rapid plant growth in response to FR light, in agreement with the recently identified synergetic effect of FR with traditionally defined photosynthetic photons (from 400 to 700 nm) on photosynthesis [37]. At the same time, FR increased LDM in leaves (Figure 1E), indicating a reduction in leaf thickness because FR did not affect SLA. Therefore, the higher photosynthesis rate was not due to the greater thickness of the leaves in response to FR.

**Figure 2:**
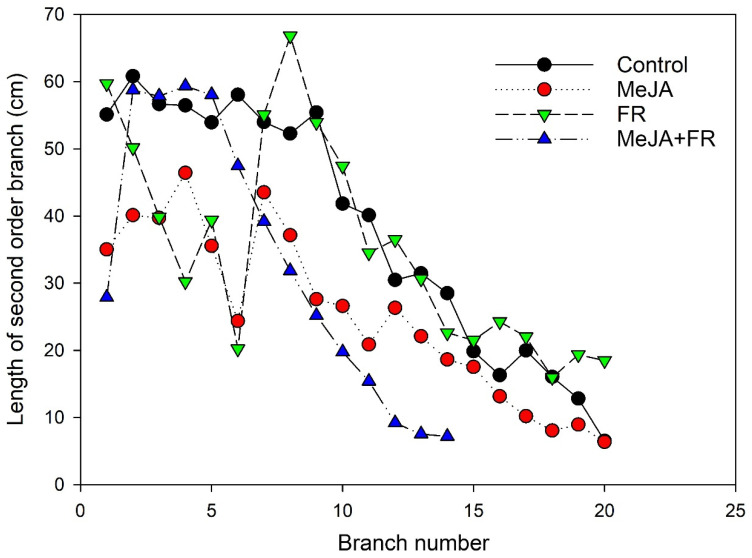
The effect of far-red light and methyl jasmonate (MeJA) application on the length of secondary order branches of *Hypericum perforatum* (*n* = 6). The branch lengths were measured at 64 DAS.

The number and length of branches are apical dominance traits and are regulated by several plant hormones [38]. A low R:FR ratio can suppress branching [39]; however, in our investigation, a significant reduction in branching was found only for the combined FR and MeJA treatment (Figure 1F). The combined application strongly reduced the length of the apical branches (Figure 2), further supporting that a combination of FR and MeJA treatment had the strongest influence on apical dominance in *H. perforatum* plants.

**Figure 3:**
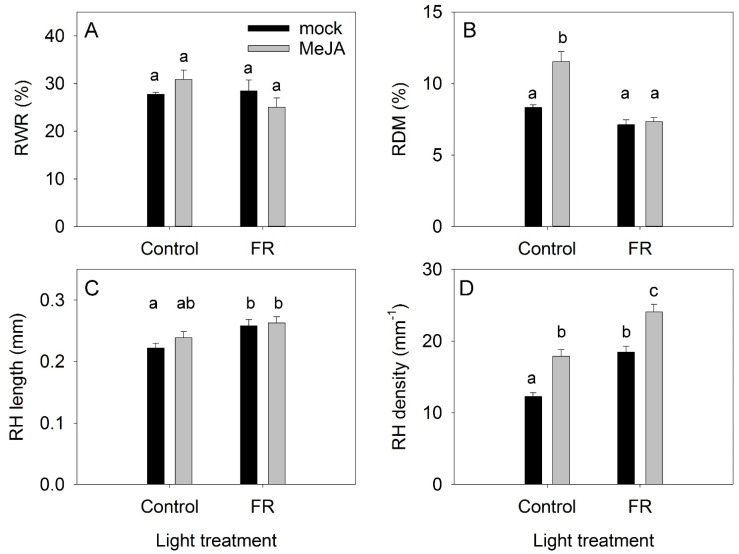
The effect of far-red light and methyl jasmonate (MeJA) application on root weight ratio (RWR) (**A**), root dry matter content (RDM) (**B**), the length of root hairs (RH) (**C**), and root hair density (**D**) in *Hypericum perforatum*. Differences between means with different letters are statistically significant. Light and jasmonate treatments were started at 36 days after sowing (DAS), and samples for measurements were taken at 65 DAS.

LWR was only weakly modulated by the MeJA and FR treatments, indicating that the allocation of dry matter to roots (RWR) was independent of either MeJA or FR treatments (Figure 3A). Under the control light conditions, MeJA treatment increased the dry matter content in roots (RDM); however, this MeJA effect was abolished under supplementary FR light (Figure 3B). FR light exposure increased the length of root hairs, whereas MeJA had no effect (Figure 3C). The root density was the lowest under the control condition, and both MeJA and FR enhanced the root hair density (Figure 3D).

### 3.2. Pigments

**Figure 4:**
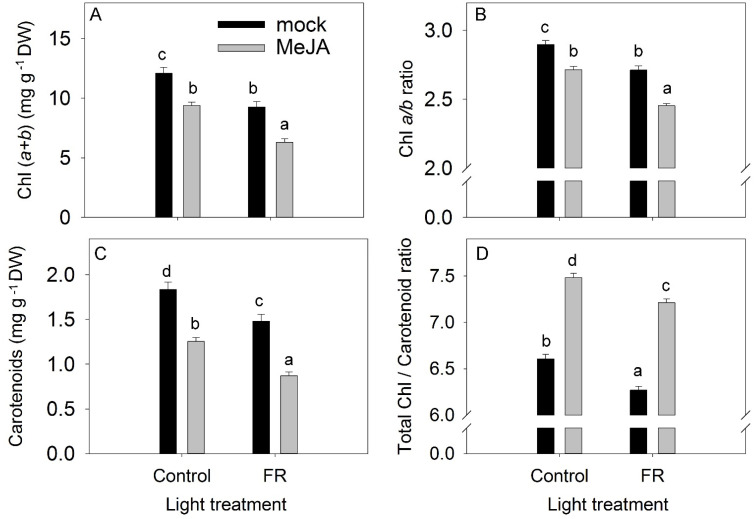
The effect of far-red light and methyl jasmonate (MeJA) application on total chlorophyll Chl (*a + b*) concentration (**A**), chlorophyll *a*/*b* ratio (**B**), carotenoid concentration (**C**), and total chlorophyll/carotenoid ratio (**D**) in *Hypericum perforatum* leaves. Differences between means with different letters are statistically significant. Light and jasmonate treatments were started at 36 days after sowing (DAS), and samples for measurements were taken at 65 DAS.

Despite the opposite effects of MeJA and FR on plant growth, both treatments reduced leaf pigment content (total chlorophyll (Chl), and carotenoids) and the Chl *a*/*b* ratio (Figure 4A–C). The chlorophyll pigment is susceptible to photodamage, whereas carotenoid pigments protect against photooxidative damage. Consequently, the Chl:carotenoid ratio is also a good indicator for evaluating the effect of environmental stress on plants [40]. In our study, MeJA application increased the Chl:carotenoid ratio (Figure 4D). The absence of an interaction between MeJA and FR in terms of the reductions in total Chl, the Chl *a*/*b* ratio, and carotenoid levels indicate that JA and FR light regulate these traits independently (Table S1).

### 3.3. Nonstructural Carbohydrates

**Figure 5:**
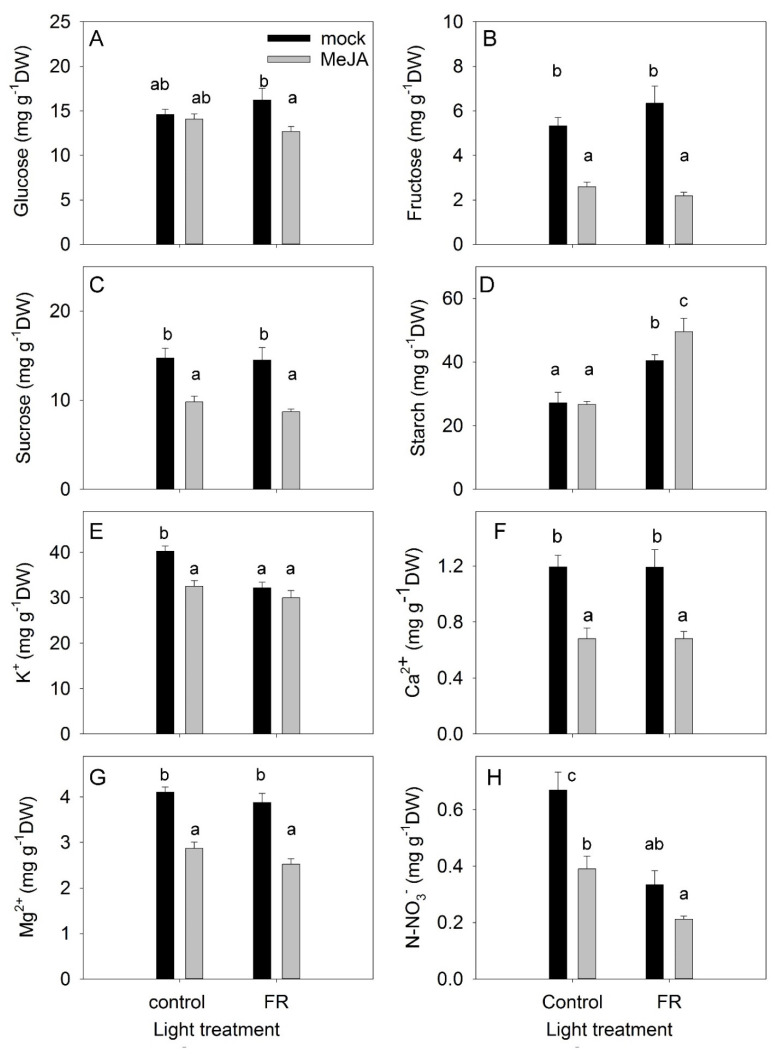
The effect of far-red light and methyl jasmonate (MeJA) application on glucose (**A**), fructose (**B**), sucrose (**C**), starch (**D**), potassium (**E**), calcium (**F**), magnesium (**G**), and nitrate (**H**) concentrations in leaves of *Hypericum perforatum* plants. Differences between means with different letters are statistically significant. *H. perforatum* seedlings were given different light and hormone treatments at 36 days after sowing (DAS). The final plant samples were taken at 65 DAS.

The accumulation of nonstructural carbohydrates in leaves is an important trait that reflects the balance between photosynthesis and the use of carbohydrates for growth processes, respiration, and synthesis of secondary compounds [41]. Under the control light conditions, MeJA treatment did not change the level of glucose in leaves; however, under supplemental FR conditions, MeJA treatment decreased the leaf glucose level (Figure 5A). Fructose and sucrose concentrations were reduced by MeJA under both light conditions, whereas the concentrations of these sugars were not affected by light conditions alone (Figure 5B,C). The reduced fructose concentration might reflect the activation of secondary metabolism by JA treatment, as fructose is the main precursor for secondary metabolites [42]. The reduced sucrose concentration might indicate a decreased source capacity of the leaves, as sucrose is the main sugar exported from the leaves to the sink organs [43].

In contrast to soluble carbohydrates, the accumulation of starch was not inhibited by MeJA (Figure 5D). Under the control light conditions, MeJA treatment did not change the accumulation of starch in leaves, but it did increase starch accumulation when provided together with FR. The enhanced starch accumulation under the combined condition indicates that growth was not restricted by the availability of nonstructural carbohydrates, but was instead limited by sink capacity (e.g., cell division and cell elongation). FR light had a positive effect on starch accumulation (Figure 5D), in agreement with studies showing that FR treatment increased photosynthesis [37].

### 3.4. Ions

The concentrations of ions in leaves indicate the uptake and transport capacity of plants. A significant interaction was found between MeJA and FR treatments in terms of the regulation of K^+^ concentrations: FR reduced the K^+^ concentration in mock plants but did not change the K^+^ concentration in MeJA-treated plants (Figure 5E). MeJA treatment decreased the accumulation of cations, with the highest impact observed for Ca^2+^. However, FR did not modulate the concentrations of Ca^2+^ or Mg^2+^ (Figure 5E–G).

The accumulation of nitrate in leaves depends on uptake and transport capacity as well as on the nitrate reductase activity in the leaves [44]. The greatest accumulation of nitrate was observed under control conditions (Figure 5H). Both FR and MeJA treatments decreased nitrate concentration and showed an additive effect when combined. Presumably, the JA effect was due to a reduction in uptake capacity (as also demonstrated for cations), while the FR effect was due to a higher photosynthesis rate and therefore more reducing power for NO_3_^−^ reduction in the leaves.

### 3.5. Total Phenolics

**Figure 6:**
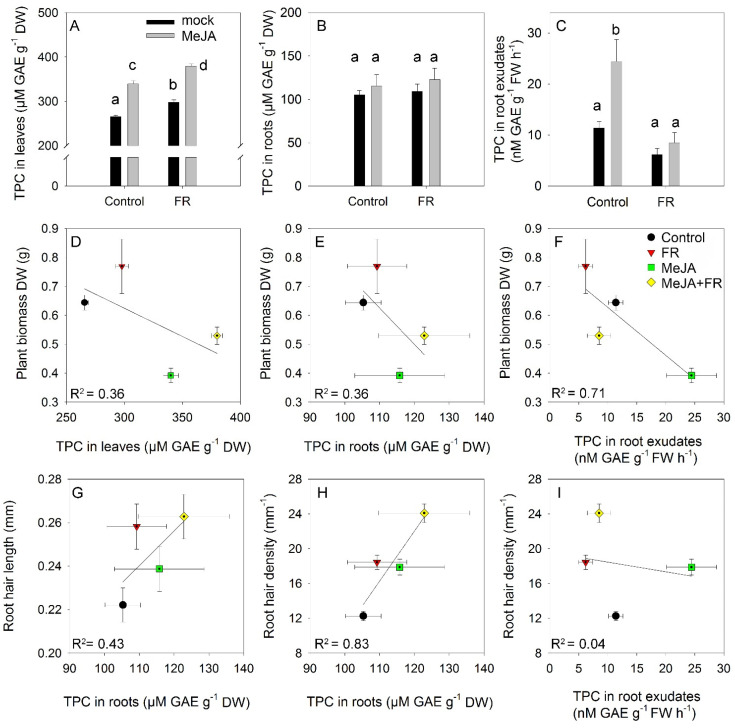
The effect of far-red light and methyl jasmonate (MeJA) application on total phenolic compounds (TPC) in leaves (**A**), roots (**B**), and root exudates (**C**) of *Hypericum perforatum* (*n* = 6). Differences between means with different letters are statistically significant. Relationships between plant biomass and TPC concentration in leaves (**D**), roots (**E**), and root exudates (**F**). Relationships between root hair length and TPC in roots (**G**). Relationships between root hair density and TPC in roots (**H**) and TPC in root exudates (**I**).

The total phenolic content is a complex trait that reflects the total antioxidant activity of cells and organs [45]. The smallest accumulation of phenolics in leaves was observed under control conditions (Figure 6A). Both FR and JA enhanced leaf accumulation of phenolics but showed no interactions, indicating a mostly independent (additive) action of these factors on the accumulation of phenolics. Roots accumulated significantly lower amounts of phenolics than leaves, and root phenolic accumulation was not affected by MeJA or FR treatments (Figure 6B). Nevertheless, MeJA treatment strongly enhanced the amounts of phenolics recovered in root exudates (Figure 6C), indicating that the rate of exudation is not directly regulated by the root phenolic concentration and that specific signals triggered by JA in the shoot can regulate the rate of phenolic exudation by the roots. Interestingly, this effect was abolished under the FR condition (Figure 6C), suggesting the existence of cross-talk between JA and FR signaling in the regulation of the amount of phenolics in root exudates.

A positive and additive effect of MeJA and FR treatments was also found for hypericin in leaves. MeJA treatment had the strongest effect on hypericin accumulation and promoted a 46% increase in its concentration, while FR light increased hypericin concentration by 16% (Table 1). MeJA treatment also enhanced the accumulation of flavan-3-ols (epicatechin, catechin, and procyanidin dimer) and coumaroylquinic acid, whereas FR light had no effect other than a decrease in the concentration of catechin, a flavan-3-ol. Several flavonols (rutin, a quercetin glycoside, and quercetin 3-glucoside) were also regulated by both MeJA and FR, but in different ways, as the concentrations of these compounds were decreased by MeJA treatment and increased by FR light.

In roots, MeJA treatment increased the accumulation of hyperfirin and other acyl phloroglucinols (APG), whereas it decreased the concentration of catechin in the opposite direction. Root exudation of hypericin and other naphtodianthrones was stimulated by MeJA treatment, but the exudation of hypericin was inhibited by FR. No interactions were detected between JA and FR (Table 1), indicating that the accumulation of all compounds was regulated mostly by independent pathways.

### 3.6. Relation between Growth Traits and Concentration of Biologically Active Compounds

The growth–defense dilemma assumes a negative correlation between growth and the accumulation of secondary compounds [3]. Therefore, we tested whether this correlation occurs in the range of our experiments where the perturbation of growth and secondary metabolism were initiated by MeJA and FR treatments. The negative correlation between biomass and leaf phenolic concentration (Figure 6D) was due to an opposite effect of JA on growth and the accumulation of phenolics; however, the same direction of FR action on biomass (Figure 1A) and phenolic concentration (Figure 6A) softened this negative correlation. The same trend was observed for the relationship between biomass and root phenolic concentrations (Figure 6E), despite the much weaker effect of JA and FR on these metabolites in the roots (Figure 6B). A stronger correlation was observed between biomass and phenolic concentrations in the root exudates (Figure 6F) due to the strong inhibitory and stimulatory effects of JA on both growth (Figure 1A) and phenolic exudation (Figure 6C).

A weak positive correlation was found between root hair length and root phenolic concentration, while a strong positive correlation was determined between root hair density and root phenolic concentration (Figure 6G,H); these correlations might reflect the high accumulation of phenolic compounds in root hairs [46]. By contrast, no correlation was found between root hair density and the amount of phenolics in root exudates (Figure 6I), indicating that phenolic exudation was independent of root hair density.

**Figure 7:**
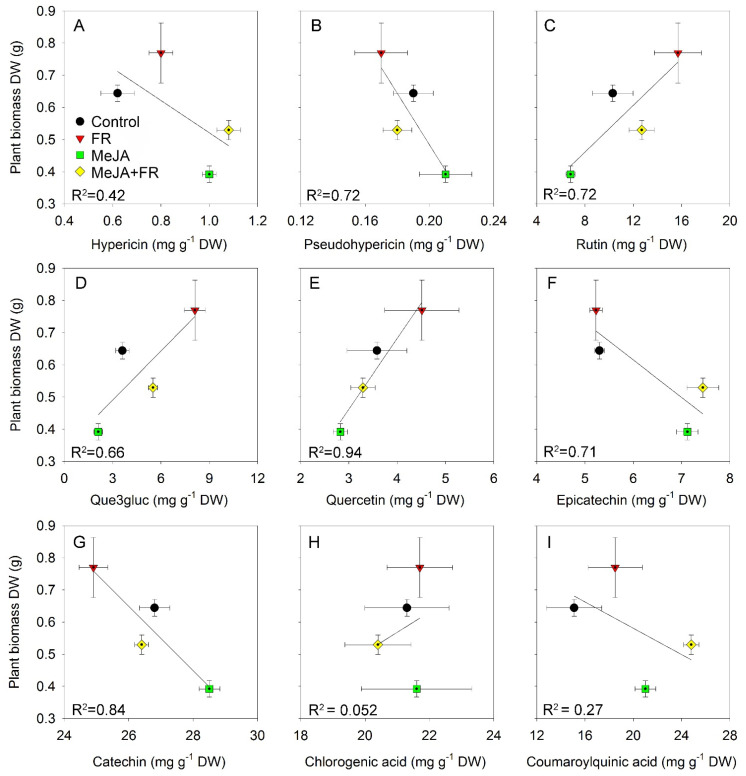
The relationship between plant biomass of *Hypericum perforatum* and hypericin (**A**), pseudo-hypericin (**B**), rutin (**C**), que3gluc (**D**), quercetin (**E**), epicatechin (**F**), catechin (**G**), chlorogenic acid (**H**), and coumaroylquinic acid (**I**) concentrations in leaves.

The different accumulation patterns for different chemical classes in response to MeJA and FR treatment (Table 1) identified different relations between biomass and the accumulation of specific compounds (Figure 7). The negative correlation between growth and hypericin concentration (Figure 7A) was due to the opposite effect of JA on growth (Figure 1A) and hypericin concentration (Table 1); however, this correlation was moderated by FR, which enhanced both plant growth (Figure 1A) and the accumulation of hypericin (Table 1). We also found a negative correlation between biomass and the accumulation of pseudohypericin (Figure 7B); however, the effect of JA and FR on the modulation of pseudohypericin concentration in leaves was not statistically significant (Table 1).

Strong positive correlations were found between biomass and flavonol content (Figure 7C–E) due to the similar effects of JA and FR on biomass and compound accumulation (Table 1). These positive correlations indicate that these treatments not only overcome the tradeoff between growth and the accumulation of biologically active compounds, but they actually increase both growth and flavonol accumulation.

A negative correlation was found for flavan-3-ols (epicatechin and catechin), which was mainly related to the diametrically opposing effect of JA on growth and the accumulation of compounds (Figure 7F,G). No correlation was found between biomass and phenolic acids, indicating that the accumulation of phenolic acids was decoupled from growth (Figure 7H,I).

**Figure 8:**
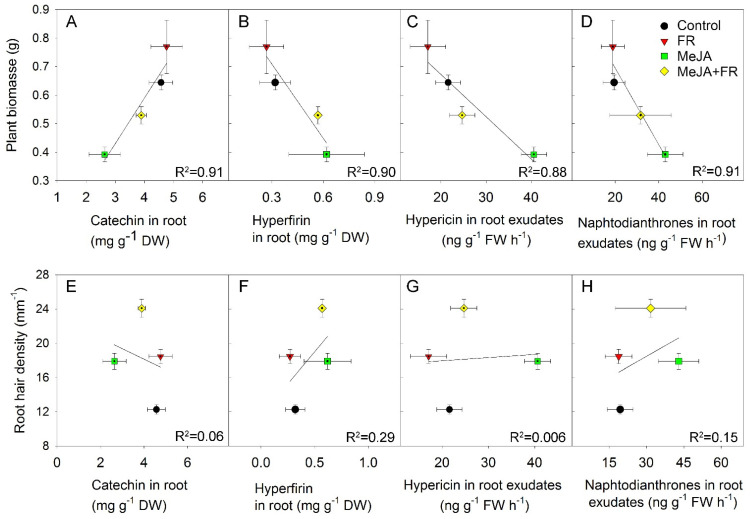
The relationship between plant biomass of *Hypericum perforatum* and catechin (**A**) and hyperfirin (**B**) concentrations in roots, as well as hypericin (**C**) and other naphtodianthrones (**D**) in root exudates. The relationship between root hair density and catechin (**E**) and hyperfirin (**F**) concentrations in roots as well as hypericin (**G**) and naphtodianthrones (**H**) in root exudates.

The type of correlation between biomass and secondary compound concentration also depended on the organ being studied. For example, despite the negative correlation noted between biomass and catechin concentrations in leaves (Figure 7G), this correlation was positive in roots (Figure 8A). This finding indicates that MeJA and FR treatments affect the distribution of this compound between the leaves and roots.

The negative correlation between plant biomass and hyperfirin concentration in roots (Figure 8B), as well as between plant biomass and the amount of hypericin (Figure 8C) and other naphtodianthrones (Figure 8D) in root exudates, were related to the opposing effects of JA and FR on plant growth and root concentration or exudation of these compounds (Figure 1A, Table 1). No significant correlation was found between root hair density and the accumulation of active compounds in roots and in root exudates, indicating that the accumulation and exudation of these compounds were independent of the development of root hairs (Figure 8E–H).

## 4. Discussion

The growth–defense hypothesis proposes that a tradeoff exists in the allocation of plant resources between growth and defense. This tradeoff is responsible for the observed inhibition of plant growth and stimulated accumulation of secondary defense/antioxidant compounds under conditions of biotic or abiotic stress. The result is a negative correlation between plant biomass and the content of secondary compounds [3]. The law of energy conservation dictates that this tradeoff cannot be completely overcome because the biosynthesis of secondary compounds requires C-skeletons and energy. However, numerous investigations have shown that the regulation of this tradeoff does not occur as a direct competition between primary and secondary metabolism for available metabolic precursors; rather, it arises through the activation of specific signaling pathways [6]. Thus, under stress conditions, the inhibition of plant growth is not primarily a result of resource limitation due to the activation of the biosynthesis of secondary metabolites; instead, it is an adaptive response driven by specific signaling pathways that allow plants to survive unfavorable stress conditions [47].

The fact that the regulation of this tradeoff occurs at the signaling level rather than at the metabolic level provides an opportunity to decouple these processes when the signaling pathways that regulate growth differ from those that trigger defense. In the present study on the medicinally important plant *H. perforatum*, we found that we could decouple growth from the accumulation of secondary compounds by simultaneous treatment of the plants with externally applied MeJA and exposure to supplemental FR light. This finding was confirmed by the full factorial experiment showing that the increased content of several types of compounds in response to JA and FR was not negatively correlated with plant biomass (Figure 7C–E,H,I). We also found a differential regulation of biologically active compounds in roots and root exudates in response to the modulation of the aboveground conditions by MeJA treatments of *H. perforatum* shoots and FR light, indicating the system regulation of secondary metabolism in the underground plant parts (Table 1).

### 4.1. Decoupling of Growth and Accumulation of Secondary Compounds

The decoupling between growth and the accumulation of secondary compounds described here by the combined treatment with MeJA and FR is consistent with several previous investigations that have shown at least a partial regulation of growth and defense responses related to the accumulation of secondary compounds by independent signaling pathways and a decoupling of these responses. The molecular basis of the growth–defense decoupling is related to the specific roles of genes from the JAZ family.

JAZs are co-receptors of COI1-associated ubiquitin ligase and repressors of JA signaling. The binding of JA to COI1 and JAZ induces JAZ ubiquitination and subsequent degradation, which relieves the promoters of JA-responsive genes [48,49]. In Arabidopsis, the JAZ family is represented by 13 members [48]. Most single *jaz* mutants show no strong phenotype, suggesting an extensive redundancy among JAZ genes; however, current evidence supports different functions and different expression patterns for different JAZs. For example, in Arabidopsis, JAZ2 is specifically expressed in guard cells and controls the stomatal response during bacterial invasion [50]. In tobacco, NaJAZi is specifically expressed in early-stage floral tissues and modulates flower-specific defenses [51]. The different functions of JAZ proteins support the hypothesis that one set of JAZ genes might be related to the inhibition of plant growth and another to the regulation of the biosynthesis of secondary compounds. This hypothesis is supported by experiments with JA agonists, which were shown to specifically activate the JAZ9 signaling pathway and induce defense responses but with no inhibition of plant growth [52]. Tobacco plants treated with a synthetic analog of JA-Ile, JA-Ile-macrolactone, also showed an enhanced defense response without compromising plant growth [53]. These examples of the upregulation of defense responses by JA agonists without affecting plant growth indicate that growth and defense regulation are independent and can be decoupled. Thus, differential regulation of a specific subset of JAZs could induce a unique subset of transcriptional factors that could trigger a defense response while allowing the maintenance of plant growth [52].

Another example of decoupling between growth and the accumulation of defense compounds was shown previously by a combination of up-regulation JA and the regulation of an additional signal pathway. Specifically, the addition of gibberellin to MeJA-treated plants restored growth without impairing the production of induced defense metabolites [54]. The basis of this decoupling is based on the direct activation of the biosynthesis of secondary metabolites through the JA-signaling pathway; however, the growth regulation occurs indirectly through the stabilization of DELLA proteins. The indirect effect of JA on plant growth regulation occurs through the inhibition of gibberellin biosynthesis, resulting in DELLA stabilization and growth inhibition [54,55]. The external addition of GA enhances DELLA degradation, thereby stimulating the growth of JA-treated plants [54].

Decoupling between growth and defense was also observed when the upregulation of the JA signaling pathway was combined with the deactivation of the phyB signaling [6]. This combination was reached in a unique Arabidopsis knockout mutant with impairment of a quintet of JAZ repressors (JAZ1/3/4/8/10) and photoreceptor phyB. The phyB mutation in this mutant recovered growth while maintaining the defense response and accumulation of an anthocyanin [6]. The mechanism by which growth and defense were decoupled in this genetic combination seems to be similar to the mechanism of decoupling observed following the activation of JA and GA signaling pathways. Indeed, the deactivation of phyB (by low R:FR ratio) increases GA biosynthesis through GA20 oxidases [56], resulting in a higher GA level. The increase in GA triggers the degradation of DELLA protein, and GA and JA antagonize via interactions between the JAZs and DELLA proteins [57].

In our study, we tried to modulate the environmental conditions to simulate the activities of signaling pathways characteristic of the mutant containing quintet JAZ and phyB mutations. To simulate the JAZ knockout mutations that continuously activate JA signaling pathways, we sprayed plants with MeJA, which enhances JAZ repressor protein degradation. To mimic phyB mutation, we supplemented the plant with FR lighting, which converts PhyB into an inactive form. Thus, we expected that the combined application of MeJA and FR light would simulate a condition where JA and phytochrome signaling pathways were activated and deactivated, respectively. We predicted that this combination of environmental factors (MeJA and FR application) would decouple growth and the accumulation of secondary compounds, and our prediction was supported by a weak or absent negative correlation between biomass and the content of total phenolics and many classes of secondary compounds (Figure 6 and Figure 7).

The FR-triggered repression of JA-mediated defense in Arabidopsis involves a stabilization of JAZs and reduced myelocytomatosis oncogene (MYC) activity. This introduces a plausible mechanistic explanation for why the jazQ-mediated defense response remains elevated in the presence of phyB, as one or more of the mutated JAZ genes in jazQ may be crucial for suppressing JA-mediated defenses when levels of active phyB are reduced [6]. Under the conditions in our experiment, the external application of MeJA might have promoted the degradation of the JAZ proteins that previously had allowed for the maintained accumulation of secondary metabolites under conditions when phyB was deactivated by supplemental FR lighting.

The decoupling effect can also be related to the form of JA applied (in our experiment, this was MeJA). MeJA was found to be more effective than JA when applied externally, which might be related to the volatile nature of MeJA [58]. MeJA is rapidly converted to JA-Ile, an active form of jasmonate [49,59]; however, exogenously applied MeJA initiated the expression of some genes that are not regulated by endogenously formed JA in barley leaves [60]. The interpretation of the effect of externally applied MeJA is complicated by the rate for the release of JA by hydrolysis and by the possible metabolism of JA to other forms that may be either biologically active or inactive [61].

Decoupling between growth and the accumulation of secondary compounds by the combined application of MeJA and FR raises the question of how JA interacts with FR to elicit its effect on the growth and accumulation of secondary compounds.

### 4.2. Cross-Talk between JA and FR and Effects on Plant Growth

Supplemental FR light caused similar growth enhancement in both the control and the MeJA-treated plants, indicating no significant interaction between JA and FR and a mostly independent action of these factors on plant growth (Figure 1A). We assume that this independence was related to (i) an interference by externally applied MeJA on the cross-talk between the jasmonate and phytochrome signaling pathways normally occurring under natural conditions and (ii) an action of FR light as a modulator of the R:FR ratio (and therefore phyB activity) as well as (iii) a source of photons for use in photosynthesis.

The cross-talk between JA and phytochrome signaling has been well demonstrated. Phytochrome signaling has been shown to affect JA activity through the regulation of JA signaling and JA biosynthesis. FR light acts on JA signaling by modulating the DELLA proteins, which act antagonistically with JAZ proteins in their plant growth and defense responses [57,62]. Deactivation of PhyB by FR light enhances the degradation of DELLA proteins [17,63]. The JAZ proteins are made more available to suppress their target transcription factors, thereby inhibiting (attenuating) the JA responses [17].

FR light suppresses the formation of bioactive JA conjugates by favoring the inactivation of hormone precursors. It also strongly upregulates ST2a [16], a gene that encodes a JA sulfotransferase that catalyzes the sulfation of hydroxyl-JA (OH-JA) to form an inactive hydrogen sulfate-JA (HSO4-JA) [64]. The activation of ST2a by FR therefore diverts the biosynthetic flux of hormone precursors away from the active JA-Ile form, thereby reducing JA signaling [65]. The externally applied MeJA is also converted to biologically active JA-Ile; however, the relatively high concentration of externally applied MeJA could overwhelm the activity of ST2a induced by FR light, resulting in the absence of an interaction between FR and MeJA.

However, the main reason why supplemental FR did not interact with externally applied MeJA might be related to the FR light action as a source of photosynthetic energy, as this process does not interact with JA signaling. Long-term experiments with supplemental FR light have shown that FR photons (700–750 nm) contributed to photosynthesis equally efficiently as traditionally defined photosynthetic active photons (400–700 nm) [37].

Indirect support for an increase in photosynthesis in response to supplemental FR can be deduced from analysis of plant growth and of nonstructural carbohydrates in leaves (Figure 5). FR treatment increased plant growth, as indicated by higher plant biomass, independent of MeJA treatment (Figure 1). However, this positive effect of FR light on *H. perforatum* growth was not related to increased dry matter allocation to the leaves or to higher SLA (Figure 1B,D), which are the typical responses to phyB deactivation [35,66]. Supplemental FR strongly increased the accumulation of starch, especially in plants treated with MeJA (Figure 5D), indicating increased source activity of the leaves. Moreover, the excess photosynthetic energy was used both for the biosynthesis of nonstructural carbohydrates and for NO_3_^−^ reduction, as indicated by the lower NO_3_^−^ concentration in the leaves of FR-treated plants (Figure 5H).

Interestingly, supplemental FR did not restore fructose and sucrose concentrations, which were reduced by MeJA treatment (Figure 5B,C). This absence of recovery does not allow us to conclude that the low concentrations of fructose and sucrose might be a limiting factor in leaf source activity. By contrast, the low concentration of fructose under the combined application of MeJA and FR indicates a high activity of secondary metabolism that utilizes fructose as a main precursor of secondary metabolites.

The inhibition of plant growth induced by JA might be related to stress response, as indicated by the decrease in total Chl level and carotenoid levels (Figure 4). The decrease in Chl levels in response to JA has been shown previously and was related to senescence-like symptoms [67]. However, the decrease in the Chl *a*/*b* ratio following JA application was opposite to the increase usually observed in response to abiotic stress [68,69]. This stress-related increase in the Chl *a*/*b* ratio might reflect Chl degradation processes, as Chl *b* may be converted to Chl *a*, thereby increasing the Chl *a* content while lowering the Chl *b* content [70]. The different response of the Chl *a*/*b* ratio to JA than to abiotic stresses might indicate a different contribution of reactions involving Chl synthesis and degradation. The response of the Chl *a*/*b* ratio to JA might depend on plant species, the administered MeJA doses, or other background environmental conditions. For example, in citrus, MeJA did not change the Chl *a*/*b* ratio [71]. In maize, MeJA did not change this ratio under control conditions but decreased the Chl *a*/*b* ratio under saline conditions [72].

The Chl:carotenoid ratio increased after MeJA application (Figure 4D), which is opposite to plant response to abiotic stress, such as drought [73], assuming that JA application initiated a stronger degradation of carotenoids than chlorophyll. A smaller accumulation of Chl under stress conditions might be attributed to impaired Chl synthesis or its accelerated degradation, or a combination of both.

The reduction in Chl content and the Chl *a*/*b* ratio observed in our experiment in response to supplemental FR light is in agreement with previous investigations on the effect of low R:FR ratios on leaf pigment content [74,75]. The change in the Chl *a*/*b* ratio under the supplemental FR condition might be related to the adjustment of the PSI/PSII ratio. PSII has a maximum absorption at 650 nm, so it does not efficiently absorb the FR light that induces plant adaptation to a low R:FR ratio by decreasing the PSI/PSII ratio to prevent an imbalance in the excitation of the two photosystems [76]. This reflects the generic plant responses for optimizing light-harvesting capacity under the specific light conditions that plants encounter. PSII has lower Chl *a*/ *b* ratio with respect to PSI, so this adjustment in the photosystems might also be responsible for the decreased Chl *a*/*b* ratio [77].

### 4.3. Cross-Talk between JA and FR and Effects on the Accumulation of Biologically Active Compounds in Plants

As with the regulation of plant growth and growth-related traits by MeJA and FR, we did not find a significant interaction between JA and FR in terms of the concentrations of the measured compounds, indicating that JA and FR regulated these compounds independently of each other (Table 1). The identification of several classes of secondary compounds that were regulated in different manners by JA and FR resulted in a different relationship between biomass and the concentration of specific compounds and indicated no common relationship between growth and the accumulation of secondary compounds. Rather, these relationships are specific and related to the classes of compounds.

**Figure 9:**
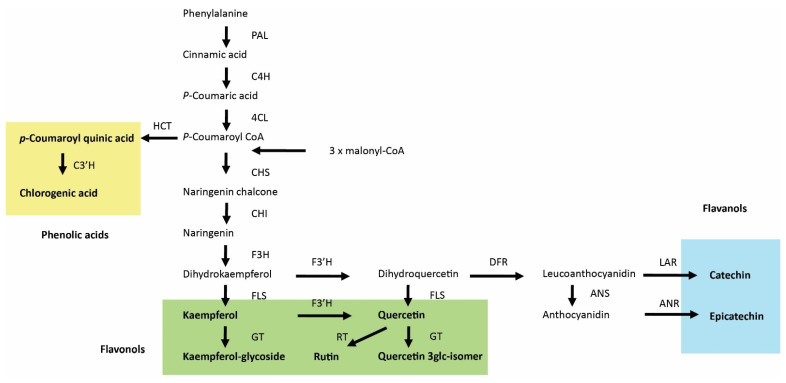
General phenolic acid and flavonoid biosynthesis in plants. PAL, phenylalanine ammonia lyase; C4H, cinnamic acid 4-hydroxylase; 4CL, 4-coumaric acid:CoA ligase; HCT, Hydroxycinnamoyl-CoA shikimate/quinate hydroxycinnamoyltransferase; C3′H, p-coumaroyl-shikimate/quinate 3′-hydroxylase; CHS, chalcone synthase; CHI, chalcone isomerase; F3H, flavanone 3-hydroxylase; F3′H, flavonoid 3′-hydroxylase; FLS, flavonol synthase; GT, glucosyltransferases; RT, rhamnosyltransferase; DFR, dihydroflavonol 4-reductase; ANS, anthocyanidin synthase; LAR, leuacoanthocyanidin reductase; ANR, anthocyanidin reductase. Phenolic acids, flavonols, and flavanols are labeled by yellow, green, and blue backgrounds, respectively.

The different patterns of accumulation of specific secondary compounds (naphthodianthrone, flavonols, flavan-3-ols, and phenolic acids) in response to JA and FR are related to their biosynthetic pathways (Figure 9). The biosynthesis of hypericin occurs through the successive condensation of small carboxylic acids [78], such as acetate and malonate, in the polyketide pathway [78,79]. Their synthesis is strictly regulated, as is the intracellular shuffling of the intermediates and final products, and their accumulation occurs in the dark glands located on different plant organs, including leaf, stem, and flower tissues [79]. Despite the independence of the polyketide pathway from the phenylalanine pathway, the accumulation was similar for hypericin and coumaroylquinic acids in response to MeJA treatment and FR light (Table 1). The stimulating effect of JA on the accumulation of hypericin agrees with studies on in vitro cultures [80,81,82]. The positive effect of FR on hypericin content might be related to an increased content of C-skeletons, which are required for hypericin biosynthesis, in response to the increased photosynthesis provided by supplemental photosynthetic FR radiation. This hypothesis is supported by previous investigations showing a positive correlation between hypericin concentration and net photosynthetic rate during the vegetative stage [79,83]. This indicates that leaves capable of high rates of CO_2_ assimilation could have a large number of dark glands, resulting in high hypericin concentrations during vegetative growth.

The other identified biologically active compounds, phenolic acids, flavonols, and flavan-3-ols, are synthesized through the phenylalanine ammonia-lyase (PAL) pathway in combination with the polyketide pathway for the flavonoids [84]. The first step in the biosynthesis of these compounds is the deamination of phenylalanine to cinnamic aid by PAL (Figure 9). Two other enzymes, cinnamate 4-hydroxylase (C4H) and 4-coumarin coenzyme A ligase (4CL), then convert cinnamic acid into p-coumaroyl CoA; at this point, the biosynthetic pathways between phenolic acids and flavonoids become separated. Enhanced accumulation of coumaroylquinic acid indicates a stimulation of the PAL pathway, which was shown to be activated by JA [85].

Up-regulation of the phenylpropanoid pathway and accumulation of phenolics are considered typical responses to JA [86] and might explain why concentrations of specific classes of secondary metabolites (i.e., flavan-3-ols and phenolic acids) were enhanced by MeJA treatment (Table 1). However, the accumulation of several compounds from the flavonol class was reduced by MeJA treatment. The biosynthetic steps leading to flavan-3-ols are downstream of flavonols and involve at least two steps. The first step is the conversion of dihydroquercetin to leucoanthocyanidin, which is activated by dihydroflavonol 4-reductase (DFR), with subsequent conversion to catechin (Figure 9). Flux analysis of catechin biosynthesis by the addition of different substrates has suggested the importance of DFR in the regulation of catechin flux and that DFR could be a regulatory element in the catechin biosynthesis pathway [87]. This flux analysis study [87] is consistent with previous results showing a need to improve the DFR requirement to enhance catechin production [88,89]. We therefore speculate that JA enhances and FR suppress the conversion of flavonols into flavan-3-ols, thereby explaining the effect of these treatments on the concentrations of the studied flavonoids.

Interestingly, the response of catechin content to MeJA and FR in shoots differed from the response in the roots, indicating a possible involvement of transport between the root and shoot in the regulation of catechin concentration in different organs. Roots of *H. perforatum* appear to be a less important organ than leaves for the accumulation of biologically active compounds (e.g., hypericin, flavonols, and phenolic acids) (Table 1), in agreement with previous investigations [90]. Roots also seem less responsive to JA and FR stimuli, based on the observed responses of total phenolics (Figure 6B). However, the root contents of catechin, hyperfirin, and other APGs were modulated by JA, indicating that systemic regulation is involved in the regulation of these compounds in roots.

In vitro studies have shown that root hair culture is a prospective production system, as root hairs might be the site where secondary compounds are accumulated [91]. We expected to find a positive correlation between the content of secondary compounds and root hair traits, such as their density and length; however, we found no such correlations (Figure 8E,F). For the compounds investigated here, the root hairs do not appear to play a key role in secondary compound accumulation.

The absence of hypericin in the roots agrees with a previous investigation [92]. Surprisingly, despite the absence of hypericin in roots, we identified hypericin in root exudates, indicating that roots can possibly synthesize but not store this compound. That roots, in theory, can be involved in hypericin biosynthesis is supported by experiments with hairy root cultures [93] and adventitious root cultures [82] that show accumulation of hypericin. The regulation of hypericin exudation by MeJA and FR was consistent with the common expectation of the regulation of root exudates by these factors: JA enhanced root exudation [94], while FR negatively affected the interaction with beneficial soil microorganisms [66], an indirect indication of a reduction in root exudation. Our approximate calculation of hypericin exudation by roots in 24 h was about 0.4% of all the hypericin present in the plants. Therefore, further optimization methods should be aimed at exploiting root exudates as important sources of secondary metabolites from plants growing in hydroponic culture.

## 5. Outlook

The standardization and high accumulation of biologically active compounds in medicinal plants are the main challenges for all herbal medicine products. These challenges can be met by selecting appropriate genotypes and optimizing the plant growth environment. The optimization of environmental conditions is straightforward for in vitro plant culture; however, this has found a relatively low level of industrial application because of the high costs. The use of standardized conditions of hydroponics in closed agriculture combines the relatively low costs of cultivation with full control of the most important environmental factors. Treatments that can overcome the growth–defense dilemma, such as FR lighting and MeJA application, are promising tools for establishing economically beneficial cultivation and allowing for the cultivation of medicinal plants with high and predictable contents of medicinally important compounds.

## Figures and Tables

**Table 1:** The influence of MeJA and FR on leaf and root contents and rates of root exudation of biologically active compounds in *Hypericum perforatum* cultivated in hydroponic system.

	Control	MeJA	FR	MeJA + FR	MeJA	FR	MeJA × FR
Leaves (mg g^−1^ DW)							
Naphthodi-Anthrones							
Hypericin	0.62 ± 0.07	1.00 ± 0.03	0.80 ± 0.05	1.08 ± 0.05	***	*	NS
Pseudohypericin	0.19 ± 0.012	0.21 ± 0.016	0.17 ± 0.016	0.18 ± 0.009	NS	NS	NS
Flavan-3-ols							
Epicatechin	5.30 ± 0.1	7.12 ± 0.22	5.23 ± 0.13	7.44 ± 0.33	***	NS	NS
Catechin	26.8 ± 0.47	28.5 ± 0.32	24.9 ± 0.44	26.4 ± 0.21	**	***	NS
Procyanidin dimer	5.06 ± 0.1	7.67 ± 0.24	5.15 ± 0.27	7.33 ± 0.38	***	NS	NS
Phenolic Acids							
Chlorogenic	21.3 ± 1.3	21.6 ± 1.7	21.7 ± 1.0	20.4 ± 1.0	NS	NS	NS
Coumaroylquinic	15.1 ± 2.3	21.0 ± 0.85	18.5 ± 2.2	24.8 ± 0.6	**	NS	NS
Flavonols							
Kaempferol-glycoside	8.3 ± 1.47	4.8 ± 0.49	11.4 ± 1.84	9.8 ± 0.36	NS	**	NS
Rutin	10.3 ± 1.7	6.8 ± 0.37	15.7 ± 1.96	12.7 ± 1.03	*	***	NS
Que-der 1	6.48 ± 0.4	4.16 ± 0.18	11.59 ± 0.74	8.70 ± 0.3	***	***	NS
Que3glc	3.6 ± 0.41	2.1 ± 0.26	8.1 ± 0.65	5.5 ± 0.28	***	***	NS
Quercetin	3.58 ± 0.62	2.82 ± 0.15	4.51 ± 0.77	3.29 ± 0.25	NS	NS	NS
Que-der 2	6.42 ± 0.87	6.44 ± 0.56	7.35 ± 0.57	7.95 ± 0.49	NS	NS	NS
Roots (mg g^−1^ DW)							
Hyperfirin	0.32 ± 0.09	0.62 ± 0.22	0.27 ± 0.10	0.57 ± 0.06	***	NS	NS
Other APGs	1.05 ± 0.07	2.05 ± 0.28	1.11 ± 0.10	1.93 ± 0.18	***	NS	NS
Catechin	4.57 ± 0.4	2.63 ± 0.54	4.76 ± 0.54	3.89 ± 0.17	*	NS	NS
Root exudates (ng g^−1^ FW(root) h^−1^)							
Hypericin	21.6 ± 2.7	40.6 ± 3.9	17.1 ± 2.8	24.7 ± 2.8	***	**	NS
Other NDA	19.5 ± 5.2	43.0 ± 8.1	18.9 ± 5.3	31.7 ± 14.1	*	NS	NS

Values are means ± SE. The data were analyzed by two-factor ANOVA. Significant effects of a factor or significant interactions between factors are labeled with asterisks: * *p* < 0.05, ** *p* < 0.01, *** *p* < 0.001. NS is not significant at the 0.05 probability level. Abbrev.: naphtodianthrones (NDA), acyl phloroglucinols (APG).

## Data Availability

Data used is within the article or Supplementary Material.

## Supplementary Materials

The following are available online at www.mdpi.com/article/10.3390/biom11091283/s1, Table S1: Analysis of variance significance levels.
