# Supplementary material for: Decoupling of Plant Growth and Accumulation of Biologically Active Compounds in Leaves, Roots, and Root Exudates of Hypericum perforatum L. by the Combination of Jasmonate and Far-Red Lighting
Source: Biomolecules. 2021 Aug 27;11(9):1283. doi: 10.3390/biom11091283 (PMC8467824; doi:10.3390/biom11091283)
Supplement: Supplementary file 1 [file biomolecules-11-01283-s001.zip › biomolecules-1335677-supplementary.pdf]

Table S1. Analysis of variance significance levels. Variables are plant biomass, leaf weight ratio (LWR), plant length, leaf dry matter content (LDM), specific leaf area (SLA), number of secondary branches (for Figure 1), root weight ratio (RWR), root dry matter content (RDM), root hair (RH) length, RH density (for Figure 3), total chlorophyll (Chl (a+b)), chlorophyll a/b ratio, total carotenoids (carot), total chlorophyll / carotenoid ratio (for Figure 4), glucose, fructose, sucrose, starch, total nitrogen (N), nitrate, K^+, Ca^{2+}, Mg^{2+} concentrations (for Figure 5), total phenolic contents (TPC) in leaves, roots, and root exudates (for Figure 6). The data were analyzed by 2-factor-ANOVA, using two light conditions (without and with far-red (FR) light) and 2 JA treatments (mock and 5 times spray with 10 µM MeJA). Means and SE are present in Figures 1, 3, 4, 5, 6, respectively. If the effect of a factor or the interaction between factors was significant, we labeled it with asterisks, * $p<0.05$, ** $p<0.01$, *** $p<0.001$. NS is nonsignificant at the 0.05 probability level.

	FR	MeJA	FR x MeJA
Figure 1			
Plant Biomass (g)	*	***	NS
LWR (%)	NS	NS	NS
Plant length, (cm)	***	***	NS
LDM (%)	***	NS	NS
SLA (cm^2 g^{-1})	NS	***	NS
Number of branches	NS	*	NS
Figure 3			
RWR (%)	NS	NS	NS
RDM (%)	***	**	**
RH length (mm)	**	NS	NS
RH density (mm^{-1})	***	***	NS
Figure 4			
Chl (a+b) (mg g^{-1} DW)	***	***	NS
Chl a/b ratio	***	***	NS
Carot (mg g^{-1} DW)	***	***	NS
Total Chl / Carot	***	***	NS
Figure 5			
Glucose	NS	*	NS
Fructose	NS	***	NS
Sucrose	NS	***	NS
Starch	***	NS	NS
Nitrate	***	***	NS
K^+	***	**	*
Ca^{2+}	NS	***	NS
Mg^{2+}	NS	***	NS
Figure 6			
TPC in leaves	***	***	NS
TPC in roots	NS	NS	NS
TPC in root exudates	***	**	*
